# The efficacy of Iranian herbal medicines in alleviating hot flashes: A systematic review

**Published:** 2016-03

**Authors:** Masumeh Ghazanfarpour, Ramin Sadeghi, Somayeh Abdolahian, Robab Latifnejad Roudsari

**Affiliations:** 1 *Department of Midwifery and Reproductive Health, Nursing and Midwifery School, Mashhad University of Medical Sciences, Mashhad, Iran.*; 2 *Nuclear Medicine Research Center, Mashhad University of Medical Sciences, Mashhad, Iran.*; 3 *Department of Midwifery, Islamic Azad University, Firuzabad, Fars, Iran.*; 4 *Evidence-Based Care Research Centre, Department of Midwifery, School of Nursing and Midwifery, Mashhad University of Medical Sciences, Mashhad, Iran.*

**Keywords:** *Herbal medicines*, *Hot flash*, *Iranian*, *Systematic review*

## Abstract

**Background:**

Hot flashes are the most common symptoms experienced by women around the time of menopause. Many women are interested in herbal medicines because of fear of side effects of hormone therapy.

**Objective:**

The aim of this systematic review was to assess the effectiveness of Iranian herbal medicines in alleviating hot flashes.

**Materials and Methods:**

MEDLINE (1966 to January 2015), Scopus (1996 to January 2015), and Cochrane Central Register of Controlled Trials (The Cochrane Library, issue 1, 2015) were searched along with, SID, Iran Medex, Magiran, Medlib and Irandoc. Nineteen randomized controlled trials met the inclusion criteria.

**Results:**

Overall, studies showed that Anise (Pimpinella anisum), licorice (Glycyrrhizaglabra), Soy, Black cohosh, Red clover, Evening primrose, Flaxseed, Salvia officinalis, Passiflora، itex Agnus Castus, Piascledine (Avacado plus soybean oil), St. John's wort (Hypericum perforatum), and valerian can alleviate the side effects of hot flashes.

**Conclusion:**

This research demonstrated the efficacy of herbal medicines in alleviating hot flashes, which are embraced both with people and health providers of Iran Therefore, herbal medicine can be seen as an alternative treatment for women experiencing hot flashes.

## Introduction

Hot flashes are the most common symptoms experienced by women around menopause time (1). Racial and cultural differences may play a role in variation of hot flashes in Western and Eastern societies (2). Hormone therapy is an effective treatment recommended for alleviating hot flashes (1, 3). In Iran, only 15% of menopausal women use hormone replacement therapy (HRT) (4, 5). 

The most frequent reason for discontinuing HRT is side effects like vaginal bleeding. There has been growing interest in natural alternatives among women in Iran, with many women efficiency accepting, safety, and lower side effects of natural therapies compared to chemical medicines (5). To our knowledge, effect of Iranian herbal medicines in alleviating hot flashes in menopausal women has not been systematically assessed. This systematic review seeks to examine the efficacy of Iranian herbal medicines in alleviating hot flashes based on the literature in this field. 

**Search Strategy**

To find relevant studies, a number of English and Persian databases such as MEDLINE (1966 to January 2015), Scopus (1990 to January 2015), and the Cochrane Central Register of Controlled Trials (The Cochrane Library, issue 1, 2015) were searched using keywords such as: hot AND (flash OR flush) AND (complementary treatments or alternative treatments or phytomedicine herbal treatments herbs evening primrose oil or St. John's wort or Hypericum perforatum or Black cohosh or cimicifuga racemosa rhizome or phytomedicine, or dong quai or Piascledine or Avacado plus or Soy or Ginseng or kava, Trigonella foenum- graecum or fenugreek or licoricered or Red clover or Evening primrose oil or yam or Flaxseed or Salvia officinalis or Vitex Agnus Castus”. In addition, SID, Iran Medex, Magiran, Medlib, Iran doc, and Google Scholar were searched in June 2014 to find equivalent keywords in Persian.

**Criteria for inclusion of studies**

1) Randomized controlled trials (RCTs) that compared oral herbs as mono/ combined preparations with control group. 2) participants included post-perimenopause with hot flashes.

**Data extraction**

Data were extracted independently by two authors and predefined checklist included age, menopausal status, sample size, duration of treatment, randomization technique, blinding method, intention- to treatment reporting, baseline comparability, outcome measures and results.

## Results

**Figure 1:**
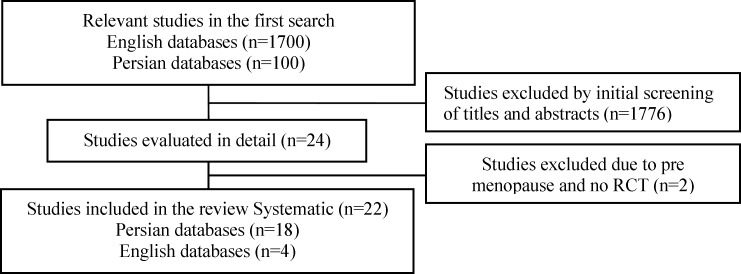
Search strategy of the study

The process of searching and selecting RCTs has been described in figure 1. In total, 19 studies were included in this systematic review. Summarized characteristics of included studies are shown in table I.

**Table I:** **C**haracteristics of 22 randomised trials included in our systematic review

**Author,** **Year**	**Duration , (Week) **	**Age (/Year)**	**Status menopause**	**Frequency hot flashes**	**Outcome **	**Drop out (%)**	**Intervention mg**	**Type of control**	**Participants** **intervention**	**Participants** **control**	**Randomization technique**	**Blinding method**	**ITI**	**Baseline** **comparability**	**Major relevant findings**
Saghafi, 2013 (6)	12	Fluoxetine/51 Black Cohosh/50	Post	>2 hot flashes frequency day	Severity of hot flash	31	Black Cohosh Capsule (containing dried roots 6.5 of black cohosh )	Fluoxetine 20 mg	29	28	Unclear	No	No	Yes	Black cohosh group (1/82±1/12) showed more improvement compared with Fluoxetine group (1/11±1/48), in regarding hot flash intensity, (p=0.08).
Shahnazi , 2013 (1).	8	T/51 P/51	Post	Symptom Vasomotor Severity≥2	Hot Flashes Frequency	0	Black Cohosh Capsule content was not known	Placebo	42	42	No	Yes	No	Yes	Results of the Repeated-Measures Analysis of Variance for Within- and Between-Groups showed significant difference
Nahidi, 2012 (8)	4	T/53 P/52	Post	Women having Experience of hot flashes	Hot Flashes Frequency and Severity	0	Capsules, Containing 330 mg of Pimpinella anisum /3 times a day	Placebo	36	36	No	Unclear	No	Yes	A statistically significant decrease in Pimpinella anisum compared with placebo group, (p<0.001)
Menati, 2013 (5).	12	Glycyrriza glabra /50 HRT/51	Peri & post	Women having experience of hot flashes	Frequancy of hot flashes	0	Glycyrriza Glabra Supplementation 1140 mg	HRT	26	26	No	Unclear	No	Yes	Glycyrriza glabra and HRT was similarly effective in alleviating frequency of hot flash but not hot flashes intensity
Nahidi, 2012 (9)	8	T/53 P/52	Post	Women having experience of hot flashes	Hot flash frequency	0	Glycyrriza glabra supplementation 1140 mg	Placebo	34	34	No	Yes	No	Yes	Glycyrriza glabra group showed statistically significant decrease in regarding hot flashes frequency and intensity compared with placebo
Abdolahi, 2006 (10).	12	T/50.1 P/51.1	Post	≥2/day	Hot flashed frequency	125	Glycyrriza glabra supplementation 250mg (containing 30-60 mg of glysesin)	Placebo	24	29	Unclear	Unclear	No	Yes	The difference between groups was not statistically significant (p<0.05).
Mirabi, 2013 (12)	4	T/51.2 P/51.7	Pre & post	Women having experience of hot flashes	Hot flashes frequency and intensity	11	Capsules containing 225 mg of valerian root /3 times a day	Placebo	35	33	Unclear	Yes	No	Yes	Hot flash frequency and intensity showed a statistical significant difference in valerian compared with placebo group
Kazemian, 2007 (11).	4 and 8 W	52/76	pre peri & post	complaint of hot flashes	Hot flashes frequency and intensity	4	Capsule valerian (containing 350 mg of valerian root /2 times a day)	Placebo	29	19	unclear	unclear	No	Yes	Valerian group showed a statistical significant decrease in regarding severity of hot flashes compared with placebo while this difference was not significant in regarding hot flash frequency
Salehi, 2013 (13).	8	T/52 P/53	Post 12	KI≥15	Hot flashes	23	Red clover Capsule (containing 45 mg isoflavones)	Placebo	28	27	Yes	Yes	No	Yes	The difference between groups was statistically significant in the 10 week of the study (p= P=0/04).
Abbaspoor , 2011 (15)	8	T/50 P/50	Post	≥3/day	Hot flash frequency and itensity	33	40 Vitagnus drop/day	Vitex agnus-castus	25	16	Yes	Yes	No	Yes	No significant difference was observed between groups.
Kazemian, 2007, (16).	12	49	Peri & post	Women having experience of hot flashes	Frequency Of hot flashes	0	Passi-pay drop ( 2 × 30 drops/day) Vitagnus droP( 2 × 30 drops/day)	Placebo	27	27	Yes	Yes	No	Yes	Vitex agnus-castus group showed a statistically significant decrease in hot flashes intensity compared with placebo. Passion Flower and vitex agnus-castus was similarly effective in alleviating hot flashes intensity
Akabari Torkestani, 2013 (21).	8	Flaxseed/50 Soy/50 Placeb Wheat flourr/50	Post	≥5/day	Hot flash frequency	0	Flaxseed Capsule 25 mg,; Soy Capsule 25 mg; wheat flour Capsule 25 mg	Wheat flour	Flaxseed/30 Soy/30	P/30	Yes	Yes	No	Yes	The comparison of hot flash frequency in the three groups using Kruskal-Wallis test showed statistically significant differences.
Baghdari, 2011 (22)	12×2 Wk, 4 WK wash out	52	Post	≥5/day	Hot flash frequency and itensity	--	Capsules containing 40 mg of Flaxseed	Placebo	23	23	No	Yes	No	Yes	Flaxseed group showed a statistically significant reduction compared with placebo in regarding hot flash intensity in (p= 0.045), however this difference was not significant between groups in regarding hot flashes frequency
Panahi, 2011 (20)	8	Piascledine /53 HRT/51	Peri ≥6	Women having experience of hot flashes	Hot flash severity Hot Flash Questions (HFQ), Visual Analog Scale (VAS)	23	Piascledine Capsule 480mg containing (Avocado oil one part, Soybean oil two parts)	HRT (0.625 mg Conjugated Estrogen tablets, plus 2.5 mg Medroxyprogesterone Acetate tablets	32	6	Yes	Yes	No	Yes	Piascledine (Avacado plus bean oil) and HRT was similarly effective in alleviating hot flashes severity(HFQ and VAS).
Hanachi, 2008 (18).	12	52	Post	Women having experience of hot flashes	Hot flash	0	The content not known	Not known	milk/15 Soy milk +exercise/12	Placebo /10	No	No	No	Not mentioned	Hot flashes decreased significantly in both soy milk and soy milk + exercise group compared with the placebo group
Abbaspoor, 2003 (19)	4	T/49 P/50	Peri	≥3/day	hot flash Intensity and frequency	40	Soy powder 50 mg containing 75 isoflavones	Casein powder	31	30	Yes	Yes	No	Yes	Hot flashes frequency and intensity showed statistical significant decrease in protein soy compared placebo group in 3 and 4 but 2 week.
Sadeghi, 2011 (23).	8	T/51 P/51	Post	≥3/day	Frequency of Hot flashes	0	daily intake of 100 mg capsules of Salvia officinalis extract	Placebo	42	42	Yes	Yes	No	Yes	The comparison between the Salvia officinalis and placebo arms was statistical significant.
Asali l, 2013 (24).	8	T/50 P/50	Post	Women having experience of hot flashes	Frequency of hot flash	5	Hypericum perforatum Capsule 480 mg (containing 990 hypercin)	passion flower 60 droups	30	30	Yes	Yes	No	Yes	A statically significant decrease in both groups (St john's wort and passion flower) at 3 and 6 week (p<0.05) compared with base line.No statistical comparison was provided between two groups.
Ghazanfarpour, 2013 (25).	8	T/53 P /52	Post	Women having experience of hot flashes	hot flash Intensity	13	Hypericum perforatum Capsule 480mg (containing 990 hypercin)	Vitex agnus-castus	31	32	Yes	Yes	No	Yes	Any significant difference was observed between two groups Hypericum perforatum. and Vitex agnus-castus
Farzaneh, 2013 (26).	6	51.9	Post	Women having experience of hot flashes	hot flash Intensity and frequency	0	evening primrose oil 1000 mg	P	33	25	Yes	Yes	No	Yes	Significant decrease on severity of hot flashes and a non-significant decrease on frequency of hot flash
Hakimi, 2004 (27).	8	T/53 P /51	Post	Women having experience of hot flashes	hot flash Intensity and frequency	8	Trigonella foenum-graecum 6 g	HRT	25	25	Unclearr	No	No	Yes	HRT showed the better effect than Trigonella foenum (Fenugreek).
Akabari Torkestani, 2013	8	Flaxseed/50 Trigonella foenum-graecum /51	Post	≥5/day	Hot flash frequency	0	Trigonella foenum-graecum 6 g	Flaxseed Capsule 25 mg,	25	25	Yes	Yes	No	Yes	A decrease from 2.20 ±0.74 to 1.31± 0.604 (40%) in Trigonella foenum and from 2± 0.74 to 0.8±0.644 (60%) in flaxseed group

**The effect of Black cohosh on hot flashes**

**Fluoxetine vs. Black cohosh**

Saghafi *et al* compared two groups of Fluoxetine and *Black Cohosh findings that both groups significantly reduced hot flash* frequency(6). Frequency of hot flash/day in Black Cohosh (1.82±1.12) decreased compared to Fluoxetine group (1.11±1.48), which was marginally statistically significant (p=0.08). 60% and 65% of patients in Fluoxetine and black Cohosh groups were satisfied with their treatment, respectively. Also, the comparison of two groups showed significant difference (p=0.04).

**Black cohosh vs. placebo**

In another trial, Shahnazi *et al* used repeated measures analysis of variance to assess the differences within and between groups (7). The frequency of hot flashes in inter-group comparison revealed a statistically significant difference in both Black Cohosh (p<0.001) and placebo (p=0.006) group across three time intervals. Comparison between groups was statistically significant at 4 (p<0.001) and 8 (p<0.001). To sum up, more clinical trial data are needed to confirm these findings.

**Effect of pimpinella anisum on hot flashes**

Nahidi *et al* assessed the effect of pimpinella anisum on hot flashes, using repeated measures ANOVA to assess the difference between and inter group differences (8). 

Gradual decrease in frequency of hot flashes was observed in Pimpinella anisum group (4.21±1.84, 3.60±1.70, 2.50±1.04, 1.63±0.80 and 1.10±0.61) and placebo group (4.24±1.87, 4.27±1.71, 4.20±1.52, 4.27±1.55 and 4.38±1.73, for five-time point’s baseline, 1, 2, 3, 4 wk respectively. Repeated measures ANOVA demonstrated statistically significant difference between 4 intervals only for Pimpinella anisum in intra group comparison (p<0.001). 

Results of t-test showed statistically significant decrease in severity of hot flash in Pimpinella anisum group compared with placebo group, (p<0.001). 11.1%, 63.9% and 25% of women suffered from severe, moderate and mild hot flashes at baseline, while it was reduced respectively to 5.6%, 69.4% and 25% at the end of study. The corresponding results in placebo group were (13.9%, 55.6 and 30.5) and (5.6%, 69.4 and 25), respectively.

**Effect of Glycyrrhiza glabra (Licorice) on hot flashes**

**Glycyrrhiza glabra vs.placebo**

Nahidi *et al* assessed the effect of Glycyrrhiza glabra (Licorice) on relief and recurrence of hot flashes (9). To detect recurrences, they interviewed with patients 1, 2, 3 and 4 wk after cessation of treatment, finding that frequency and severity were similar in both groups at baseline. Repeated measures ANOVA regarding the frequency of hot flashes in inter-group comparison revealed a statistically significant difference in Licorice group across eight time intervals, though this difference was not significant in placebo group. 

Glycyrriz aglabra group showed statistically significant decrease in hot flashes frequency compared to placebo group in 8intervals, 1 (p<0.002), 2 (p<0.001), 3 (p<0.001), 4 (p<0.001), 5 (p<0.001), 6 (p<0.001), 7 (p<0.002), and 8 (p<0.001) wk. At the baseline, 22.2%, 46.6% and 31.1% of women suffered severe, moderate and mild hot flashes, while it was reduced to 2.3%, 33.3% and 64.4% at the end of study, respectively.

Corresponding results for placebo group were (24.4%, 40% and 35.5) and (22.3, 33.3 and 44.4). As indicated by repeated measures ANOVA, hot flash severity reduced significantly from 1-8 wk in Glycyrriza glabra group while for Placebo group, this reduction was significant only for 1 wk. Also, 2 wk after cessation of treatment, women reported significant relief of hot flashes frequency and intensity.

Another trial by Abdolahi *et al* showed gradual decrease in frequency of hot flashes in both Glycyrriza glabra group (6±2.8, 3.95±2.86, 2.66±1.68 and 1.06±1.19) and placebo group (4±2.4, 2.92±2.52, 3.5±2.62 and 2.38±2.59) (10). After 12 wk, only Glycyrriza glabra- at the baseline, after 4.08 and 12 wk of stud treated patients experienced statistically significant reduction (p<0.05) compared to the baseline. After 12 wk, the reduction in frequency of hot flashes in Glycyrriza glabra-treated patients was more significant than placebo group. 

**Glycyrriza glabra (Licorice) vs. HRT**

Menati *et al* reported statistically significant decrease in frequency of hot flashes in HRT (p=0.008) and a non-significant decrease in Glycyrriza glabra group (p=0.157), however, the comparison between groups showed no significant difference (p=0.134) (5). Hot flashes severity reduced significantly in HRT group (p=0.031), but this reduction was not significant in Glycyrriz aglabra group (p=0.698) and the comparison between two (p=0.019). In fact, Glycyrriza glabra group was not significantly different from HRT with respect to frequency of hot flashes, but latter experienced more effective reduction in hot flash intensity. To sum up, based on three trials discussed above, it seems that Glycyrrizaglabra (Licorice) has alleviating effect on hot flashes, though further studies are needed to support the current evidences.

**Effect of valerian on hot flashes**

Kazemian *et al* found significant decrease in the frequency of hot flashes in period between baseline and 4 (p<0.05) or 8 (p<0.01) wk after trial in Valerian group (11). However, Valerian and placebo groups were not compared. The comparison of two groups in terms of severity of hot flash by Mann-Whitney test showed statistically significant difference after 8 wk (p<0.01), but this was not case after 4 wk. Another trial by Mirabi *et al* showed statistically significant decrease in intensity and frequency of hot flashes in Valerian group (p<0.001) while placebo group remained unaffected (12). Also, comparison of two groups showed a statistical significant difference at 4^th^ and 8^th^ wk of study (p<0.001). It seems that Valerian group can remarkably alleviate severity of hot flashes, though more studies are needed to support current evidences.

**Effect of red clover on hot flashes intensity**

Salehi *et al* and Ehsanpour *et al* assessed the effect of red clover on hot flashes intensity (13, 14). According to Friedman test, frequency of mild, moderate and severe hot flash decreased significantly compared to baseline in both red clover (p<0.001) and placebo groups (p<0.001). Mann-Whitney test showed statistically significant decrease between groups at wk 10 the 10^th^ wk of study (p=0.04), but it was not significant at at 2^nd^ and 4^th^ wk. In a meta-analysis of six trials about effect of red clover, a subgroup analysis was conducted to determine most effective dose of red clover in frequency decreasing of hot flashes (15). Pooled effect size was larger in trials in which red clover was administrated at a dose of 80 mg -0.79 (-2.35 to 0.78) followed by a dose of 40 mg -0.40 (-2.33 to 1.53) and 160 mg -0.30 (-5.54 to 4.94). It seems that higher dose of red clover might be more effective.

**Eeffect of Vitexagnus-castus on hot flashe**

**Vitexagnus- castus vs. placebo**

Study by Abbaspoor *et al* showed progressive decline in frequency of hot flashes in Vitexagnus-castus (6±2.58, 4±2.52, 2±2.38, 1.28±2.26 and 0.76±2.16) and placebo group (5.94±2.2, 6±2.34, 5.81±2.40, 5.44±2.42 and 4.75±2.84) at baseline, 2, 4 and 6 wk after trial (16). More statistically significant decrease in hot flashes frequency was also observed in Vitexagnus- castus group after 2 (p=0.015), 4 (p=0.012), 6 (p>0.001) and 8 (p>0.001) wk compared to placebo groups. Also, reduced severity of hot flashes in women receiving Vitexagnus-castus was more significant than placebo group after 2 (p=0.015), 4 (p>0.001), 6 (p>0.001) and 8 (p>0.001) wk.

**Vitexagnus- castus vs. Passionflower**

Another study by Kazemian *et al* which compared three groups (Passion Flower, Vitexagnus-castus and placebo), found statistically significant decrease in hot flashes intensity 2 and 4 wk after trial in Passion Flower group (17). However, no statistically significant decrease was observed between baseline and 2^nd^ wk. Significant decline was observed between baseline and 2^nd^ or 4^th^ wk in Vitexagnus-castus group. No statistically significant difference, nevertheless, was observed between 2^nd^ and 4^th^ wk. The comparison of three groups by Kruskal-Wallis test showed statistically significant difference between them after a 30-day period, but it was not significant after 15 days. Mann-Whitney test was used to determine the difference between groups, and it indicated that mean change of Vitexagnus-castus was significantly higher than placebo group. 

Passion Flower group and vitexagnus- castus group were similarly effective in alleviating hot flashes intensity. Therefore, it appeared that Vitexagnus-castus and Passion Flower is was significantly different from placebo. In a duplicate trial in 2010, Kazemian et al assessed the effect of Passion Flower on hot flash frequency (18). A gradual decrease in the frequency of hot flashes in Passion Flower group (7.26, 5.48 and 4.52) and placebo group (38.81, 23.07 and 21.7) was reported 2 and 4 wk after trial beginning . Significant decrease was observed in hot flashes frequency after 2 or 4 wk in both groups, the decrease was significance between 2^nd^ and 4^th^ wk only in Passion group. No statistical comparison between Passion Flower and placebo was provided. In sum, vitexagnus-castus has had significantly alleviating effect on hot flashes, though further studies are needed to support these evidences.

**Effect of soy on hot flashes**

**Soy vs. placebo**

Hanachi *et al* divided patients randomly in three groups of soy milk, soy milk plus exercise and placebo (19). Reduction of hot flashes in both soy milk (72%) and soy milk +exercise groups (83%) was significantly higher than placebo group. Another study by Abbaspour showed a gradual reduction in frequency of hot flashes in protein soy (10.38±3.38, 9.43±3.13, 7.17±2.38, 5.45±1.74) and placebo groups (10.41±2.76, 10.91±3.22, 9.94±2.84, 9±2.54) at the baseline, after 2, 3 and 4 wk of study (20). The difference between groups was observed after 2 wk (p=0.06), 3 wk (p<0.001), and 4 wk (p<0.001). This was not the case of baseline (p=0.973). The corresponding findings about hot flashes intensity were (28.9±11.06, 25.41±10.31, 16.76±6.48, 9±2.75 and 9±2.75) for the protein soy group, and (29.16±9.07, 30.22±10.17, 26.97±8.75, and 24.69±7.66) for the placebo group at the baseline (p=0.92), after 2 (p=0.07), 3 (p<0.001), and 4 wk (p<0.001) of the study. 

**Piascledine (Avacado plus soybean oil) vs. HRT**

Panahi *et al* divided the participants into two groups: Piascledine (Avacado plus soybean oil) and HRT menopausal (21). The severity of hot flashes was measured using two different methods, hot flash questionnaires (HFQ) and visual analog scale (VAS) of hot flash severity. Former contained four questions: (the length of hot flash, the impact of hot flashes on waking up, the interference of hot flash with daily activities and possibility of having night sweat. The latter is a horizontal line graded from 0-100 (0=no hot flash and 100=unbearable hot flash). Piascledine (Avacado plus soybean oil) and HRT were similarly effective in alleviating hot flashes severity, (length of hot flash (p=0.796), the impact of hot flashes on waking up (p=0.111), the interference of hot flash with daily activities (p=0.949) and possibility of having night sweat (p=0.671). Also, VAS showed 23.57 points reduction in Piascledine group compared to a 16.21-points decrease in HRT group (p=0.800). 

**Comparison of soybeans and flaxseed with wheat flour control group**

The comparison of three groups based on Kruskal-Wallis test showed statistically significant difference between groups in a 30-day period. Akbari Torkestani *et al* divided the participants randomly into three interventions, soybeans, flaxseed, and wheat flour groups (22). According to Kruskal-Wallis test, the intensity and frequency of hot flashes were similar in all three groups at baseline. There was not any statistically significant difference with respect to hot flash intensity between three groups at 4^th^ wk (p=0.485) and 8^th^ wk (p=409). The comparison of three groups based on Kruskal-Wallis test showed significant difference between groups at 8^th^ wk of the trial, but this was not the case at 4^th^ wk. There was statistically significant decrease in hot flash frequency only in soy group. To sum up, it seems that soy was more effective in alleviating hot flashes, though further studies are needed to support these evidences. 

**Effect of flaxseed on hot flashes**

The effect of flaxseed on hot flashes was also evaluated by Baghdarin *et al* (23). They conducted a double-blind, randomized, cross- over study (with a wash out period of two wk) on two groups of flaxseed and placebo for six wk. Flaxseed group showed a significant reduction compared to placebo group with respect to hot flash intensity (p=0.045), but this difference was not significant with regard to hot flashes frequency. Therapeutic effect was only significant in women experiencing 5-7 cases of daily hot flashes (p<0.001). In contrast, Akbari Torkestan *et al* did not find any significant decrease in frequency and intensity of hot flashes, which was probably due to low dose administration (25 mg vs. 40 mg) (22). Again, more studies are needed to clarify whether high dose (40 mg) is more effective than low dose (25 mg).

**Effect of Salvia officinalis on hot flashes**

Sadeghi *et al* found the significant effects of Salvia of ficinalis extract and placebo on the frequency of hot flashes (24). Although the comparison of Salvia officinalis and placebo groups was significant, still further studies are needed to confirm these evidences. 

**Effect of St. John’s wort (hypericum perforatum) on hot flashes**

**St. John’s wort vs. passion flower**

Asali *et al* showed a progressive decrease in intensity of hot flashes in passion flower (8.1, 5.6 and 4) and St. John's wort group (9.3, 5.8 and 4.4) at the baseline, after 3 and 6 wk of study (25). Significant decrease was observed in both groups (St. John’s wort and passion flower) at 3^rd^ and 6^th^ wk (p<0.05) compared to baseline. Although 60% and 27.5% of women suffered severe hot flashes in St. John's wort and passion flower groups respectively, it was reduced to 10% and 3.4% at 6^th^ wk (the end of study).

**St. John’s wort vs. vitexagnus-castus**

Ghazanfarpour *et al* found significant decrease in both groups (St. John's wort and vitexagnus-castus), but this difference was not significant between flower and vitexagnus-castus groups after 1 (p=0.98) and 2 months (p=0.68) (26).

**Effect of evening primrose oil on hot flashes**

In a trial on effect of evening primrose oil on hot flashes, frequency of hot flash decreased from 5.2±1.9 to 3.2±1.8 in the evening primrose and from 5.4±1.9 to 3.7±20 in placebo group (27). The severity of hot flash reduction was greater than placebo group, but, the difference between groups was not significant (p=0.23). The severity of hot flashes changed from 5.9±1.5 to 3.4±1.4 (-2.6±1.60) in evening primrose and from 5.9±1.7 to 4.1±2.0 (-1.8±1.2) in placebo group, which was statistically significant. In conclusion, it seems that evening primrose oil is more effective in alleviating hot flashes. Again, more studies are needed to confirm the current results.

**Trigonella foenum-graecum (fenugreek) on hot flashes**

Hakimi *et al* divided the patients randomly into two groups of trigonella foenum and HRT (28). A gradual decrease was observed in Trigonella foenum (7.08±0.596, 4.36±0.53 and 2.60±0.46) and HRT group (7.47±0.71, 1.72±0.34, 0.84±0.23) at the baseline, after 4 and 8 wk of study. The frequency of hot flashes decreased significantly Trigonella foenum group, at wk 4 and wk 8 of the study compared baseline. That is, the effectiveness of HRT group was greater than Trigonella foenum group.

**Trigonella foenum**

Another trial by Akbari Torkestani *et al* showed a decrease from 2.20±0.74 to 1.31±0.604 (40%) in Trigonella foenum and from 2±0.74 to 0.8±0.644 (60%) in the flaxseed group (29). The comparison of two groups was significant at 8^th^ wk. To sum up, it seems that Trigonella foenum may be effective in alleviating hot flashes.

## Discussion

To our knowledge, this is the first systematic review about therapeutic effect of Iranian herbal medicine on hot flashes. Herbal medicine plays a key role in treatment of many diseases. Both Iranian people and health providers are interested in herbal medicine. Overall, studies have shown that Pimpinella anisum, licorice (Glycyrrhiza glabra), soy, black cohosh, red clover, evening primrose, Pimpinella anisum, Flaxseed, Salvia officinalis, Passi-Vitagnus, Piascledine (Avacado plus soybean oil), St. John's wort (Hypericum perforatum), passion flower and Valerian may have alleviate side effects of hot flashes.

**Determining the suitable wash-out period for cross-over design**

Nahidi *et al* conducted several interviews with women at 1, 2, 3 and 4 wk after cessation of treatment to detect recurrence of hot flashes (30). Women, who reported significant relief 2 wk after therapy cessation, suggested that effects of phytoestrogen in licorice can persist even 2 wk after cassation of treatment. This finding may help determine the sufficient wash-out periods essential between periods of a crossover design. However, it should not be generalized to other phytoestrogens, as Baber *et al* showed that effects of phytoestrogen in red clover persisted one wk after cassation (2). 

**Clinically treatment effect**

To assess the treatment satisfaction threshold, Wyrwich *et al* used Menopause symptoms treatment satisfaction questionnaire (MS-TSQ) (31). This questionnaire was designed by Hill *et al* to assess satisfaction of women with level of menopausal symptoms treatment over 4 levels of treatment with desvenlafaxine. It is composed of 7 items on menopausal symptoms, including hot flashes, sweats night, sleep, mood, libido, concentration ability, medication tolerability along with one overall question about treatment satisfaction. Each item is rated on scale of 0-4, which includes “extremely dissatisfied,” “dissatisfied,” “neutral,” “dissatisfied,” and “extremely satisfied,”. The treatment satisfaction threshold is difference between average reductions in two mentioned symptoms for women who reported were “neutral” and “satisfied” about treatment.

Hill *et al* used only two of 7 items (hot flash and sweat) to measure women’s satisfaction reduction of 1.64 in hot flashes is considered as clinically meaningful threshold with respect to 50% placebo effect (32). It is important to note that they determined the treatment satisfaction threshold based on 50% of placebo effect. Future studies are needed to focus on determining the threshold of treatment satisfaction base on low effect of placebo. Also, future studies should be taken into account both statistical and clinical significance.

**Low placebo effect**

Several factors may involve in placebo response, including doctor-patient relationship, patients' positive or negative expectations of treatment, cultural factors like patients' perception of colors, forms, and drug names, along with their experience and perception of fate and faith are involved in this process (33, 34). This systematic review showed that placebo had a slight effect on alleviating hot flash. One possible explanation for this can be cultural difference. Future research can use mixed method designs with semi-structured interviews and open-ended questions such as RCTs to explore why some participants show low responses to placebo. 

**Suggestion for future trials**

Many studies have shown beneficial effects of herbal medicine in decreasing hot flashes. Future studies can compare the effectiveness of herbal medicines with HRT groups. Further studies are required to measure biological parameters of estrogen, including estradiol, estron, Follicle-stimulating hormone (FSH) and Sex hormone-binding globulin (SHBG) to investigate the relationship between biological parameters and intensity and frequency of hot flashes. 

**Limitations**

The weak methodology of many studies used in our systematic review can be one of the potential limitations of this study. Small sample sizes, inadequate treatment allocation, lack of intention to treatment report, unclear blinding method and unmentioned randomization technique can degrade the validity of the results. 

## Conclusion

This research demonstrated the efficacy of herbal medicines in alleviating hot flashes, which are embraced both with people and health providers of Iran (5, 35). Therefore, herbal medicines can be considered as an appropriate alterative for women experiencing hot flashes. 

## Conflict of interest

Authors have no conflict of interests.
